# A New Year Greeting

**Published:** 1887-01

**Authors:** 


### Editorial.

### A NEW YEAR GREETING.

In entering upon another year THE JOURNAL desires to extend to every reader its wishes for a Happy New Year. Not in the perfunctory spirit of a mere "compliment of the season" do we say this. The best measure of the sincerity of a man's wishes lies in the earnestness of his endeavor to effect their accomplishment. Without such endeavor the best of wishes are but mere hypocrisy, or at least thoughtless idle-talk. Therefore, when THE JOURNAL wishes its readers a Happy New Year, it is but giving to them its promise that as far as lies in its power it will strive to make the year a happy, interesting and profitable one to them.

We hold to the somewhat old-fashioned idea that happiness lies only in the performance of good, honest, conscientious work toward the fulfillment of some worthy end. To the practitioner of medicine it lies in earnest effort for the relief of suffering humanity about him and in equally earnest effort for the advancement of medical science, thereby contributing indirectly to the still wider relief of the sufferings of those who shall come after him. No man can throw his heart and soul into such work without reaping a reward in the shape of happiness, and for a large part of such work no other reward can be looked for. Yet, after all, labor of this sort is not gratuitous. It is but paying with interest to the succeeding generation the debt which is due to the one which has gone before. It may be in the consciousness of having discharged a just obligation that the satisfaction of such work is found.

To the furtherance of work of this character THE JOURNAL will continue, as in the past, to lend its influence and its example. To encourage all that is truly worthy of a noble profession, to discard all that tends to degrade and debase that profession, to promote true happiness by inspiring a spirit of earnest, conscientious work — such shall be its aim.

It is in this sense that THE JOURNAL wishes its readers a Happy New Year.

